# Demographics and Health Behavior of Video Game and eSports Players in Germany: The eSports Study 2019

**DOI:** 10.3390/ijerph17061870

**Published:** 2020-03-13

**Authors:** Kevin Rudolf, Peter Bickmann, Ingo Froböse, Chuck Tholl, Konstantin Wechsler, Christopher Grieben

**Affiliations:** Institute of Movement Therapy and movement-oriented Prevention and Rehabilitation, German Sport University Cologne, Am Sportpark Muengersdorf 6, 50933 Cologne, Germany; p.bickmann@dshs-koeln.de (P.B.); froboese@dshs-koeln.de (I.F.); c.tholl@dshs-koeln.de (C.T.); k.wechsler@dshs-koeln.de (K.W.); c.grieben@dshs-koeln.de (C.G.)

**Keywords:** eSports, gaming, video games, physical activity, sleep, nutrition, training, health promotion

## Abstract

The number of video game and eSports players is steadily rising. Since little is known about their health behavior to date, the present study examines the demographics and health behavior of video game and eSports players. In this cross-sectional study, data on demographics, health status, physical activity, nutrition, sleep, and video game usage were assessed via a web-based survey of *n* = 1066 players (91.9% male; 22.9 ± 5.9 years; body mass index (BMI): 24.6 ± 4.8 kg/m²) in Germany in 2018. The majority of respondents (95%) reported a good to excellent health status. Two thirds (66.9%) engaged in moderate to vigorous physical activity for more than 2.5 h/week. The average duration of sitting and sleep time was 7.7 ± 3.6 h/day and 7.1 ± 1.3 h/day, respectively. Mean fruit and vegetable consumption was 2.7 ± 1.8 portions/day. Video games were played for 24.4 ± 15.9 h/week on average. Partial Spearman correlations revealed poor positive associations of video game play time to sedentary behavior (rho = 0.15; *p* < 0.01) and BMI (rho = 0.11; *p* < 0.01), as well as a poor negative association to self-reported health status (rho = −0.14; *p* < 0.01). These results indicate the good subjective health of this target group. Nevertheless, the high amount of video game play time and its poor negative association to health status indicate a need for specific health promotion strategies for this target group.

## 1. Introduction

Video games and competitive gaming—what is known as eSports—in which two or more human players compete in video games with a defined set of rules [1], are becoming increasingly popular recreational activities with a growing popularity in society. Big eSports events like *The International* attract thousands of spectators on site and millions of viewers online from all over the world [2]. The number of eSports enthusiasts, i.e., people that play or watch eSports regularly, is estimated to be 198 million worldwide [3], the number of regular video game players is even higher [4].

While the social interest in gaming and eSports is steadily rising, academic research on those involved in the phenomenon is relatively new [5]. To date, most research pertains to the fields of informatics, media, business, and sociology [5]. Many studies focus, among other things, on whether eSports can be considered a sport [6,7,8], on the economic scope of gaming and eSports [9,10] as well as on the psychological effects of gaming on brain function [11,12,13] and character traits [14,15,16]. In this context, the focus on gaming addiction has increased [17,18,19], with the inclusion of internet gaming disorder in the Diagnostic and Statistical Manual of Mental Disorders (DSM-5) in 2015 [20] and gaming disorder in the International Classification of Diseases and Related Health Problems (ICD-11) in 2018 [21]. In addition, the first studies on eSports performance have emerged, focusing mainly on the cognitive skills and challenges eSports players face [22,23,24]. In terms of research on health and health behavior, however, further research is needed. Besides the demographics of this target group, additional information about health behavior would be useful to evaluate the need for specific health-promotion strategies. 

Prolonged screen time, accompanied by long periods of sedentary behavior are recognized as risk factors for numerous chronic diseases [25,26,27] and all-cause mortality [26,27,28,29]. Since gaming and eSports, by their very nature, require long periods of sedentary screen time, it stands to reason that such players pose a high-risk group in exceptional need of health promotion. As envisioned in the German Prevention Act of 2015 [30], gaming itself could offer new ways of gaining access to and providing health promotion to the target group in their specific setting. For this purpose, however, data on the current situation of video game and eSports players in terms of health status and health behavior are necessary. Hence, the aim of the present study is an assessment of the demographics and health behavior of video game and eSports players. In addition, possible associations between video game play time and health behavior are examined.

## 2. Materials and Methods 

### 2.1. Study Design and Setting

The present study was conducted as a cross-sectional online survey. The web-based questionnaire was distributed on the project website [31], social media (*Facebook*, *Discord*, *Instagram*), on different eSports and gaming forums, and in person at eSports events (*ESL One Cologne*, *Gamescom*). In addition, authors’ personal contacts to eSports teams and organizations were used to further disseminate the study. Recruitment messages included the aim of the investigation as well as information on eligibility criteria and incentives to participate (chance to win one of three €50 *Amazon* gift cards). Data collection took place from July 5th to October 7th 2018. 

The study was approved by the ethical committee of the German Sport University Cologne (reference: 053/2018).

### 2.2. Participants

Participants were eligible for the present study if they were currently living in Germany, understood German and engaged in eSports and/or gaming. Before starting the questionnaire, a participant information page was displayed online and consent was obtained. Additionally, a filter question regarding the country of residence was displayed. If a residence outside Germany was stated, the questionnaire ended and the participant was excluded.

### 2.3. Measures

The questionnaire was administered via the online survey tool *Unipark* (*Questback* GmbH, Cologne, Germany). It was developed to address different aspects of demographics, health, health behavior (physical activity, sleep, nutrition) and video game usage as well as participants’ personal opinion on the effects of nutrition, sleep, and physical fitness on eSports performance. Since filter questions were used, the number of questions ranged between 18 and 26. 

The following demographic data were collected: participants’ age, gender, level of education, and employment status. The wording and rating of these questions were designed in accordance with the standards of the German Federal Statistical Office [32]. 

Questions on health behavior comprised types of sports activities (semi-open-ended questions, multiple responses possible), duration of moderate to vigorous physical activity (4-point rating scale: “less than 1 h per week”, “1–2.5 h per week”, “2.51–5 h per week”, “more than 5 h per week”), duration of sedentary behavior (hours per day), duration (hours per night) and quality of sleep (4-point rating scale: “very good”, “quite good”, “quite poor”, “very poor”), and daily fruit and vegetable consumption (portions per day). In addition, one item on subjective health status (5-point rating scale: “excellent”, “very good”, “good”, “not so good”, “poor”) was included. Body mass index (BMI) was calculated using self-reported body weight and height.

Data on video game usage comprised average play time (hours per week), prioritized game title (semi-open-ended question) and training practices. The latter included a filter question as to whether participants engage in regular eSports training that is conducted to improve specific skills. If the answer was yes, further questions on training organization were given (multiple choice: “training in a (regional) association with a coach”, “training in a (regional) association without a coach”, “training in a team with a coach”, “training in a team without a coach”, “training with friends”, “training alone and/or with random opponents/players”), along with training time (hours per week), percentage of training that takes place at the PC/console (percentage of overall training), and training content were given. For training content, a matrix table question with possible content was presented (“game mechanics” (learning about the strengths and weaknesses of the playable characters and how to control them), “tactics (rehearsing tactics)”, “game analysis (of own games)”, “game analysis (of competitors or role models)”, “team building (for team games only)”, “communication with team members (for team games only)”, “responsiveness”, “targeted training of fine motor skills/precision/’mechanical skill’”, “dealing with stressful situations (during the game)”, “physical fitness”, “relaxation/recovery”, “other”); possible responses were “always”, “often”, “sometimes”, “never”, “not applicable/no details given/don’t know”.

Moreover, participants were asked to classify themselves as professional players (regularly earning significant revenue from eSports (prize money, sponsors, salary from clubs)), amateurs (playing eSports but without earning a significant amount of money), regular players (playing video games or eSports more than once a week, but without taking part in official tournaments and leagues), occasional players (playing video games or eSports several times a month or less, and without taking part in official tournaments and leagues) or non-players. The latter were excluded from all analyses. Occasional players were excluded from all analyses regarding video game usage.

In the last part of the questionnaire, participants were asked for their personal opinion on how balanced nutrition, sufficient sleep, and good physical fitness affect eSports performance (5-point scale: “very positive”, “quite positive”, “neither”, “quite negative”, “very negative”).

After a pilot study, small changes regarding wording and rating scales were made and the resulting questionnaire was distributed.

### 2.4. Statistical Analyses

Descriptive statistics were performed on all questions. Numerical data were described by means and standard deviations, ordinal data by medians and frequencies, and nominal data by frequencies. 

Differences between groups (player status) were examined by the Kruskal–Wallis test, ANCOVA and Fisher’s exact test.

To examine possible associations between health behavior and video game play time, partial Spearman correlations were calculated. The resulting coefficients were interpreted as no correlation (rho = 0–0.09), poor (rho = 0.10–0.29), fair (rho = 0.30–0.59), moderately strong (rho = 0.6–0.79), very strong (rho = 0.8–0.99), and perfect correlation (rho = 1) [33].

The significance level for all analyses was set at *p* < 0.05. All statistical analyses were conducted using IBM SPSS Statistics 25 (IBM Corp., Armonk, NY, USA).

## 3. Results

### 3.1. Participants

In total, 3488 people opened the first page of the online survey, 1272 (36.5%) proceeded to the next page, and 1112 (31.9%) completed the full questionnaire. We excluded 33 participants for not living in Germany, 10 for not engaging in eSports or gaming, and 13 for inconsistent data (e.g., age compared to employment status). The resulting sample of 1066 (30.6%) participants consisted of 14 current and 33 former professional players, 355 amateurs, 577 regular players and 87 occasional players (see Table 1).

### 3.2. Demographic Data

Table 1 displays the characteristics of the total sample and all player status groups. The predominantly male (*n* = 980; 91.9%) sample is, on average, between 20 and 30 years old and well-educated. While more than half of the sample consists of apprentices (*n* = 158; 14.8%), pupils (*n* = 162; 15.2%) and university students (*n* = 292; 27.4%), about a third of all respondents is working full-time (*n* = 348; 32.6%). Regarding the BMI, about half (*n* = 550; 51.3%) of the sample can be classified as normal weight (*n* = 112 (10.5%) participants did not provide data on height or weight). 

While education and BMI are similar across all groups, some statistically significant differences between groups are present regarding age, gender distribution and employment (see Table 1; effect sizes for all significant differences can be found in Supplementary File 1). 

### 3.3. Health Behavior 

The vast majority of the sample has an excellent (*n* = 194; 18.2%), very good (*n* = 407; 38.2%) or good (*n* = 412; 38.6%) self-reported health status. Only 4.8% (*n* = 51) report a poor and 0.2% (*n* = 2), a very poor health status. 

**Figure 1:**
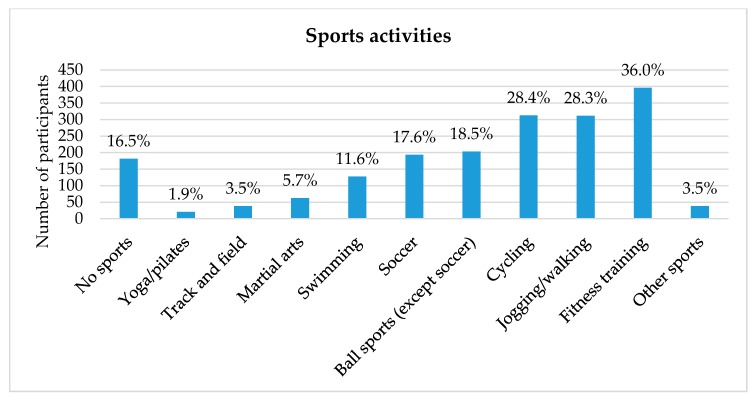
Sports activities of sample participants. Multiple responses were possible (n = 1100).

Regarding physical activity, the sample is engaged in a variety of sports activities (see Figure 1). This goes in line with participant responses regarding the duration of moderate to vigorous physical activity: Two thirds (*n* = 713; 66.9%) of the sample engage in moderate to vigorous physical activity of more than 2.5 h a week. The average amount of sedentary behavior is 7.7 ± 3.6 h a day.

Across all player status groups, the median sleep quality is “quite good” (“very good”: *n* = 227; 21.3%, “quite good”: *n* = 642; 60.2%, “quite bad”: *n* = 181; 17.0%, “very bad”: *n* = 16; 1.5%). The average sleep duration is 7.1 ± 1.3 h per night. 

Mean fruit and vegetable consumption is 2.7 ± 1.8 portions/day and the recommendation of five portions a day is fulfilled by 11% (*n* = 117) of the total sample.

No statistically significant differences between player status groups regarding the mentioned parameters of health behavior were found, except for the difference that professional players (7.8 ± 1.3 h/night) sleep significantly longer than former professional players (6.7 ± 1.4 h/night; *p* = 0.04; see Table 2).

### 3.4. Video Game Usage

The prioritized game titles of the sample are displayed in Table 3. The most popular games are tactical shooters (e.g., *Counter-Strike: Global Offensive, PlayerUnknown’s Battlegrounds*) and multiplayer online battle arenas (MOBAs; e.g., *League of Legends, Dota 2*).

Table 4 displays the average play time and training characteristics of the sample (occasional players excluded). Focusing on players who participate in official tournaments or leagues, more than half (*n* = 231; 57.5%) engage in structured training and 67 of these (29.0%) have a coach. The average training time of those who engage in structured training is 15.4 ± 6.5 h/week for professional players, 18.6 ± 13.8 h/week for former professional players, 12.1 ± 10.2 h/week for amateurs, and 7.8 ± 8.7 h/week for regular players.

**Figure 2:**
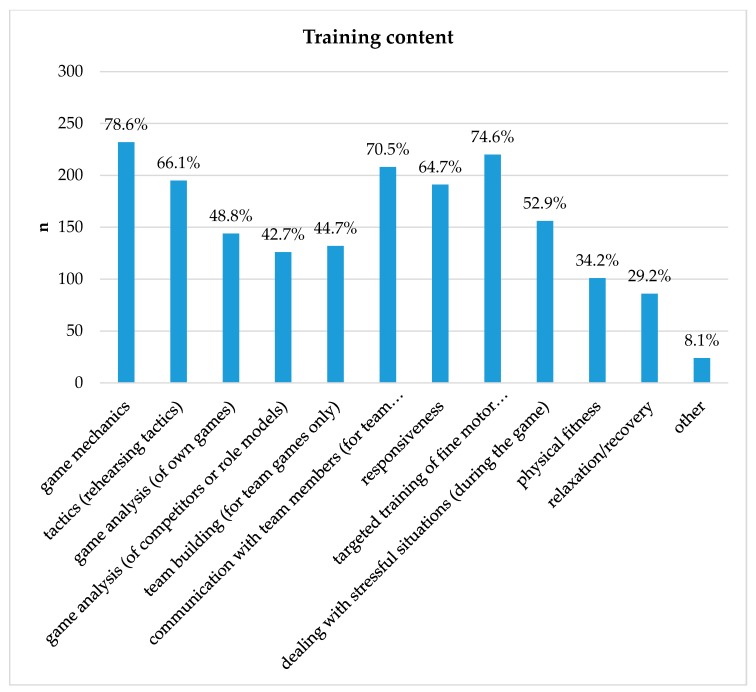
Content of players’ structured trainings (*n* = 295). Values represent respondents who stated that they always train or often train for that aspect in their training sessions.

The content that is “always” or “often” implemented in the structured training sessions is displayed in Figure 2.

### 3.5. Respondents’ Opinion on Influencing Factors of eSports Performance

The majority of respondents believe good physical fitness brings very positive (*n* = 343; 32.2%) or quite positive (*n* = 513; 48.1%) effects to eSports performance. The results are similar regarding balanced nutrition (“very positive”: *n* = 231; 21.7%; “quite positive”: *n* = 521; 48.9%) and sufficient sleep (“very positive”: *n* = 756; 70.9%; “quite positive”: *n* = 227; 21.3%).

### 3.6. Associations between Health Behavior and Video Game Play Time

Controlling for age, gender, and education, partial Spearman correlations reveal poor positive associations between video game play time and sedentary behavior (rho = 0.15; *p* < 0.01) and BMI (rho = 0.11; *p* < 0.01). In addition, controlling for age, gender, education and BMI, a poor negative association can be observed between video game play time and self-reported health status (rho = −0.14; *p* < 0.01). This association is still present after additionally controlling for sedentary behavior (rho = −0.12; *p* < 0.01).

No statistically significant or relevant (rho < 0.10) associations are found between video game play time and sleep parameters, physical activity, and fruit and vegetable consumption. 

## 4. Discussion

### 4.1. Key Results

The aim of the present study was to examine the demographic and gaming characteristics of video game and eSports players, as well as their health-related behaviors. In total, data of more than 1000 predominantly young, male, and well-educated video game and eSports players were collected and analyzed. The results show the self-reported health status of the target group is mostly very good, but in terms of health behavior, some room for improvement remains. 

In contrast to non-competitive gaming, where the gender distribution is almost equal [34,35], the present study confirms other research which shows that the vast majority of eSports players is male [10,36]. This may be due to the more competitive character of eSports in comparison to regular gaming. Prior research has shown that eSports’ so-called “core-genres” (such as tactical shooters, fighting games, or sport games) are more strongly favored by men [37]. The eSports scene in terms of community and game design is strongly influenced by men and a male perspective, respectively [37,38]. This may tend to predominantly attract male players and, in this way, lead to a vicious circle. Additionally, sexism, offensive language and online harassment are reoccurring problems in some eSports titles [38,39,40,41] that might further deter female players. 

The results show that the composition of the target group is different from the general population: In line with results from previous studies on gaming and eSports [42,43], the present sample includes predominantly young and well-educated participants. While about half of the sample is still in some kind of educational process, e.g., school or university students, the other half is working part-time or full-time. The question may arise as to whether time spent participating in eSports just exacerbates people’s sedentary behaviour at school, university or the office. However, it should also be considered that eSports, or gaming in general, may offer a possibility to get access to a target group—adolescents and young adults—that is too often overlooked by traditional health campaigns [44]. For example, promoting the implementation of trained coaches could be a possible approach to integrate holistic and health-promoting training concepts into the daily routines of eSports players. Although research identifying the essential skills for success in eSports is limited, it may be assumed that fitness training and training content from traditional sports may also benefit skills that are necessary for good eSports performance like, e.g., sustained attention [45] or reaction time [46,47]. Right now, about half of the respondents, and predominantly those who compete in official tournaments and leagues, complete structured training but most of it takes place without a coach and almost solely in the digital environment. Bringing these digital skills into the real world by means of practicing specific physical exercises could reduce sedentary behaviour and bring real health benefits [48,49,50]. In this respect, it can be seen as a very positive result that classical strength and endurance exercises are quite frequently represented in this target group. However, only one-third of the sample associates fitness training with the content of a structured eSports training. It seems that the respondents do engage in fitness training but not for the purpose of improving their eSports performance.

Only a minority of eSports players does not participate in any sport activity at all, and almost two-thirds of the present sample achieve the WHO’s global recommendation on physical activity for adults [51]. This is a far bigger portion than in the average German population where only 42.6% (females) and 48.0% (males) meet the same recommendation [52]. Nevertheless, there is much room for future improvement, particularly when one considers the young age of the sample group and how physical activity tends to inevitably decline over the average human life span [53,54]. 

The average play time (of more than 20 h per week) in all player status groups shows the importance of gaming in each respondents’ life. After long hours at work, school or university, three to four hours of additional gaming result in high amounts of sedentary behavior, which is known to be a risk factor for non-communicable diseases [25,26,27,28,29]. The observed very weak inverse associations of video game play time and self-reported health status, BMI and sedentary behavior are at least pointing in the same direction. However, due to the cross-sectional design of the study, the direction of causality remains unclear.

In terms of sleep duration and quality, it is notable that the average sleep duration is lower than that of a comparable group of healthy adults [55] and one in six respondents reports that they do not sleep well. This raises questions about the reason for these results. A possible explanation may be connected to the use of screen media, which is known to have adverse effects on sleep duration [56,57] and sleep quality [56,58]. Although no general association of video game play time and sleep quality was found in the data, more specific associations between media use and sleep cannot be generally ruled out by the present data. Previous studies indicated that the use of light-emitting devices before bedtime is connected to increased sleep disturbances [59,60,61,62]. Similar associations may also apply to video game usage if, for example, a too short period between media use and bedtime exists. Since we did not collect data on these parameters, a more detailed analysis was not possible. Hence, further research on this topic within the target group of video game and eSports players is needed.

While the overall self-reported health status of the sample is very good, the BMI reveals that only half of the sample can be classified as being of normal weight. The percentage is similar to that of the average German population (45.3%, [63]) but lower than that of people of similar age (18–30 years: 72.3–59.9% classified as normal weight [63]). Even though the BMI does not provide information on body composition, it might hint at another area in which health promotion is needed: diet. With only 11% of the sample fulfilling the recommendation of the German Nutrition Society of five portions of fruit or vegetables a day [64], the nutrition of eSports and video game players seems as equally unbalanced as in the average German population [65]. The low fruit and vegetable intake in the present sample might be connected to the previously mentioned long video game playing time and is in line with results from Shang et al. [66] that observed lower fruit and vegetable consumption in people with high screen time. Since more information on the consumed food and drinks was not collected in this study, it stands to reason that the lack of fruit and vegetables may be compensated by other (less healthy) food or dietary supplements. Further research in this area is needed to obtain a comprehensive picture of video game and eSports players’ specific dietary behavior, and to identify possible potential for improvement—not only in regard to health but also in relation to performance. 

A good sign for the possible improvement of the health situation and health behavior is the result indicating that the vast majority of video game and eSports players support the statements that fitness, sleep and nutrition have positive effects on their eSports performance. As mentioned above, supporting these beliefs by further research and the development of specific concepts may help to get access to, and implement and maintain, healthy and holistic lifestyles in this target group. 

### 4.2. Limitations

Although the present study provides a useful insight into the characteristics and health behaviors of video game and eSports players, it also has inherent limitations. First, for the purpose of providing information on a wide range of different health behaviors, most topics could only be addressed through a small number of questions, some of them created for the specific purpose of this study (e.g., the classification of player status). Complete versions of validated questionnaires would have allowed more detailed analyses and comparisons to previous research. However, the aim was to obtain a comprehensive overview of the target sample but still keep the questionnaire short and feasible. In the future, further studies with emphases on certain behaviors are needed to build upon this initial, mostly descriptive, data.

Second, since the targeted sample belongs to a special population, recruiting took place in areas where we believed video game and eSports players are best represented. By collecting data on eSports events, players of the respective games that were shown there might be overrepresented in the present sample. To attenuate this bias, the online survey was posted on a variety of gaming websites and forums. However, about half of the sample still represents players of just one game—*Counter Strike: Global Offensive*—although the share of this game regarding overall players is lower [67]. Moreover, the number of players in each player status group differs extensively. Although this might limit the power of the statistical analyses, we decided to keep the professional players in the analyses to provide insight on this very specific and hard to reach subgroup of eSports players. 

Third, the present study solely relies on self-reported data. Hence, the results might be affected by social desirability [68] as well as by over- or under-estimation of certain behaviors like physical activity [69,70]. To minimize these effects, we excluded inconsistent data and checked the data for extreme values to prevent distortion through (deliberately) false information (“trolling”). All cases with inconsistent data were discussed between the authors before a decision about exclusion was made. In addition, the online survey was programmed so that individual health behaviors exceeding 24 h per day were not possible. However, effects of social desirability and inaccurate reporting cannot generally be excluded. Future studies should consider integrating social desirability scales [71] or using objective data to control for such effects.

Lastly, the results show that the current sample does not represent the average population in terms of demographic characteristics (e.g., age or gender distribution). Hence, the comparisons must be treated with caution.

## 5. Conclusions

Overall, the results of this research project indicate that the health and lifestyle needs of video game and eSports players are not unique to this target group. They face the same challenges regarding a healthy lifestyle as the average population does. Nevertheless, against the backdrop of the (weak but relevant) detrimental associations between video game playing time and health outcomes, the importance of providing new ways of health promotion to this target group is indisputable. Even more so, considering the rising popularity of eSports as well as the increasing digitalization of society, inevitably bringing even more sedentary lifestyles. Changing the form of game training (which up to now, has been predominantly digital) and embedding it in the real world, might be a possible way to integrate physical activity and other health-related behaviors into the training routines of video game and eSports players.

## Figures and Tables

**Table 1:** Sample characteristics.

Group	Age ^1^ [years] Mean (SD)	Gender ^2^ [“male”] *n* (%)	BMI ^3^ [kg/m²] Mean (SD)	Education ^2^ [“higher education entrance qualification or higher”] *n* (%)	Employment ^2^ [“full-time employment”] *n* (%)
**Total sample** (*n* = 1066)	22.9 (5.9)	980 (91.9)	24.6 (4.8)	556 (52.5)	348 (32.6)
**Professional players** (*n* = 14)	22.9 (5.0)	12 (85.7)	23.1 (4.9)	5 (35.7)	1 (7.1) ^F,R,O^
**Former professional players** (*n* = 33)	26.1 (5.8)^A,R^	33 (100.0) ^O^	26.0 (5.2)	16 (48.5)	17 (51.5) ^P,A,R^
**Amateurs** (*n* = 355)	22.1 (5.0)^F,O^	342 (96.3) ^R,O^	25.0 (5.1)	162 (51.3)	106 (29.9) ^F,O^
**Regular players** (*n* = 577)	22.8 (6.1)^F^	521 (90.3) ^A,O^	24.4 (4.6)	307 (53.2)	188 (32.6) ^P,F^
**Occasional players** (*n* = 87)	25.0 (7.6)^A^	72 (82.8) ^F,A,R^	24.6 (4.2)	46 (52.8)	36 (41.4) ^P,A^
**Sig.**	< 0.01	< 0.01	0.08	0.07	< 0.01

^1^: Kruskal–Wallis test; ^2^: Fisher’s exact test; 3: ANCOVA (controlling for age and gender). Superscript letters indicate statistically significant differences (*p* < 0.05) to other groups in the same column: ^P^—professional players; ^F^—Former professional players; ^A^—Amateurs; ^R^—Regular players; ^O^—Occasional players.

**Table 2:** Data on health behavior of the sample.

Group	Health Status [mode] *n* (%)	Physical Activity [mode] *n* (%)	Sedentary Behavior [hours/day] Mean (SD)	Sleep Time [hours/night] Mean (SD)	Sleep Quality [mode] *n* (%)	Fruit and Vegetable Consumption [portions/day] Mean (SD)
**Total sample** (*n* = 1066)	“good” 412 (38.6)	“2.51–5 h per week” 357 (33.5)	7.7 (3.6)	7.1 (1.3)	“quite good” 642 (60.2)	2.7 (1.8)
**Professional players** (*n* = 14)	“good” 6 (42.9)	“more than 5 h per week” 7 (50.0)	7.3 (3.3)	7.8 (1.3)^F^	“quite good” 7 (50.0)	3.5 (2.2)
**Former professional players** (*n* = 33)	“very good” 13 (39.4)	“2.51–5 h per week” 10 (30.3)	7.6 (3.9)	6.7 (1.4)^P^	“quite good” 15 (45.5)	3.0 (2.2)
**Amateurs** (*n* = 355)	“good” 146 (41.1)	“2.51–5 h per week” 126 (35.5)	8.0 (3.7)	7.2 (1.3)	“quite good” 208 (58.6)	2.6 (1.7)
**Regular players** (*n* = 577)	“very good” 228 (39.5)	“2.51–5 h per week” 191 (33.1)	7.7 (3.6)	7.0 (1.3)	“quite good” 361 (62.6)	2.7 (1.8)
**Occasional players** (*n* = 87)	“very good” 39 (44.8)	“more than 5 h per week” 29 (33.3)	7.0 (3.1)	7.2 (1.1)	“quite good” 51 (58.6)	2.8 (1.4)
**Sig.**	0.90	0.84	0.34	0.04	0.02*	0.46

Kruskal–Wallis test. Superscript letters indicate statistically significant differences (*p* < 0.05) to other groups in the same column: ^P^—professional players; ^F^—Former professional players. * no statistically significant post-hoc difference after adjusting for multiple testing.

**Table 3:** Prioritized game titles of the sample (n = 1066).

Game Title	Frequency *n* (%)
*Counter-Strike: Global Offensive*	522 (49.0)
*League of Legends*	157 (14.7)
*PlayerUnknown’s Battlegrounds*	51 (4.8)
*Fortnite—Battle Royale*	50 (4.7)
*FIFA* Series	45 (4.2)
*Dota 2*	43 (4.0)
*Overwatch*	24 (2.3)
*Rainbow Six Siege*	21 (2.0)
*Rocket League*	19 (1.8)
*World of Warcraft*	12 (1.1)
Other games *	122 (11.5)

* Games with less than 10 nominations were summed up as “other games“.

**Table 4:** Data on video game play time and training characteristics.

Group	Play Time ^1^ [hours/week] Mean (SD)	Structured Training ^2^ [“yes”] *n* (%)	Form of Training [mode] *n* (%)	% of Overall Training Time that Takes Place at the PC/console ³ Mean (SD)
**Total sample** (*n* = 978) *	24.4 (15.9)	295 (30.2)	“training with friends” 133 (45.1)	90 (16.8)
**Professional players** (*n* = 14)	28.6 (12.0)	8 (57.1) ^R^	“training alone and/or with random opponents/players” 4 (50.0)	91.0 (11.1)
**Former professional players** (*n* = 33)	25.3 (20.1)	17 (51.5) ^R^	“training in a team without a coach” 7 (41.2)	84.2 (21.6)
**Amateurs** (*n* = 355)	28.4 (16.6) ^R^	206 (58.4) ^R^	“training in a team without a coach” 97 (47.1)	89.4 (13.8)
**Regular players** (*n* = 576)	21.7 (14.7) ^A^	64 (11.1) ^P,F,A^	“training with friends” 47 (73.4)	80.7 (22.4)
**Sig.**	<0.01	<0.01	-	0.12

“Form of training” (multiple responses possible) and “Percentage of overall training time that takes place at the PC/console” were only answered by players participating in structured training. * Occasional players (*n* = 87) did not answer questions regarding video game usage, one missing data set; ^1^: ANCOVA (controlling for age, gender and BMI); ^2^: Fisher’s exact test; ^3^: Kruskal–Wallis test; Superscript letters indicate statistically significant differences (*p* < 0.05) to other groups in the same column: ^P^—professional players; ^F^—Former professional players; ^A^—Amateurs; ^R^—Regular players.

## Supplementary Materials

The following are available online at https://www.mdpi.com/1660-4601/17/6/1870/s1, Table S1: Effect sizes of the statistically significant differences regarding demographics and health behavior.

## References

[1] German Esports Federation ESBD Was ist eSport? Definition [What is esport? Definition]. https://esportbund.de/esport/was-ist-esport/.

[2] Esports Charts The International 2018. https://escharts.com/tournaments/dota2/international-2018.

[3] Newzoo 2017–2022 Global Esports Audience Growth. https://newzoo.com/key-numbers.

[4] Newzoo Global Games Market Report: Free Version. https://newzoo.com/insights/trend-reports/newzoo-global-games-market-report-2019-light-version/.

[5] Reitman J.G., Anderson-Coto M.J., Wu M., Lee J.S., Steinkuehler C. (2019). Esports Research: A Literature Review. Games and Culture.

[6] Willimczik K. (2019). eSport “ist” nicht Sport–eSport und Sport haben Bedeutungen [eSport “is” not sport—eSport and sport have meanings. An analysis from the perspective of a philosophy of language instead of ontological debates]. Ger. J. Exerc. Sport Res..

[7] Parry J. (2019). E-sports are Not Sports. Sport Ethics Philos..

[8] Holden J.T., Kaburakis A., Rodenberg R. (2017). The Future Is Now: Esports Policy Considerations and Potential Litigation. J. Legal Asp. Sport.

[9] Ströh J.H.A. (2017). The eSports Market and eSports Sponsoring.

[10] GlobalWebIndex eSports Trends Report 2018. https://cdn2.hubspot.net/hubfs/304927/Downloads/Esports-report.pdf?t=1528372092399.

[11] West G.L., Konishi K., Diarra M., Benady-Chorney J., Drisdelle B.L., Dahmani L., Sodums D.J., Lepore F., Jolicoeur P., Bohbot V.D. (2018). Impact of video games on plasticity of the hippocampus. Mol. Psychiatry.

[12] Stanmore E., Stubbs B., Vancampfort D., de Bruin E.D., Firth J. (2017). The effect of active video games on cognitive functioning in clinical and non-clinical populations: A meta-analysis of randomized controlled trials. Neurosci. Biobehav. Rev..

[13] Shams T.A., Foussias G., Zawadzki J.A., Marshe V.S., Siddiqui I., Müller D.J., Wong A.H.C. (2015). The Effects of Video Games on Cognition and Brain Structure: Potential Implications for Neuropsychiatric Disorders. Curr. Psychiatry Rep..

[14] Bányai F., Griffiths M.D., Király O., Demetrovics Z. (2019). The Psychology of Esports: A Systematic Literature Review. J. Gambl. Stud..

[15] Greitemeyer T., Mügge D.O. (2014). Video games do affect social outcomes: A meta-analytic review of the effects of violent and prosocial video game play. Pers. Soc. Psychol. Bull..

[16] Granic I., Lobel A., Engels R.C.M.E. (2014). The benefits of playing video games. Am. Psychol..

[17] Gentile D.A., Bailey K., Bavelier D., Brockmyer J.F., Cash H., Coyne S.M., Doan A., Grant D.S., Green C.S., Griffiths M. (2017). Internet Gaming Disorder in Children and Adolescents. Pediatrics.

[18] González-Bueso V., Santamaría J.J., Fernández D., Merino L., Montero E., Ribas J. (2018). Association between Internet Gaming Disorder or Pathological Video-Game Use and Comorbid Psychopathology: A Comprehensive Review. Int. J. Environ. Res. Public Health.

[19] Paulus F.W., Ohmann S., von Gontard A., Popow C. (2018). Internet gaming disorder in children and adolescents: A systematic review. Dev. Med. Child Neurol..

[20] Petry N.M., Rehbein F., Ko C.-H., O’Brien C.P. (2015). Internet Gaming Disorder in the DSM-5. Curr. Psychiatry Rep..

[21] World Health Organization International Statistical Classification of Diseases and Related Health Problems. https://icd.who.int/browse11/l-m/en.

[22] Himmelstein D., Liu Y., Shapiro J.L. (2017). An Exploration of Mental Skills Among Competitive League of Legend Players. Int. J. Gaming Comput. Med. Simul..

[23] Railsback D., Caporusso N., Ahram T.Z. (2018). Investigating the Human Factors in eSports Performance. Advances in Human Factors in Wearable Technologies and Game Design, Proceedings of the AHFE 2018 International Conferences on Human Factors and Wearable Technologies, and Human Factors in Game Design and Virtual Environments, Held on July 21–25, 2018, in Loews Sapphire Falls Resort at Universal Studios, Orlando, Florida, USA.

[24] Pedraza-Ramirez I., Musculus L., Raab M., Laborde S. (2020). Setting the scientific stage for esports psychology: A systematic review. Int. Rev. Sport Exerc. Psychol..

[25] Bailey D.P., Hewson D.J., Champion R.B., Sayegh S.M. (2019). Sitting Time and Risk of Cardiovascular Disease and Diabetes: A Systematic Review and Meta-Analysis. Am. J. Prev. Med..

[26] Biswas A., Oh P.I., Faulkner G.E., Bajaj R.R., Silver M.A., Mitchell M.S., Alter D.A. (2015). Sedentary time and its association with risk for disease incidence, mortality, and hospitalization in adults: A systematic review and meta-analysis. Ann. Intern. Med..

[27] Patterson R., McNamara E., Tainio M., de Sá T.H., Smith A.D., Sharp S.J., Edwards P., Woodcock J., Brage S., Wijndaele K. (2018). Sedentary behaviour and risk of all-cause, cardiovascular and cancer mortality, and incident type 2 diabetes: A systematic review and dose response meta-analysis. Eur. J. Epidemiol..

[28] Chau J.Y., Grunseit A.C., Chey T., Stamatakis E., Brown W.J., Matthews C.E., Bauman A.E., van der Ploeg H.P. (2013). Daily sitting time and all-cause mortality: A meta-analysis. PLoS ONE.

[29] Rezende L.F.M., Sá T.H., Mielke G.I., Viscondi J.Y.K., Rey-López J.P., Garcia L.M.T. (2016). All-Cause Mortality Attributable to Sitting Time: Analysis of 54 Countries Worldwide. Am. J. Prev. Med..

[30] (2015). Gesetz zur Stärkung der Gesundheitsförderung und der Prävention (Präventionsgesetz–PrävG) [Act to Strengthen Health Promotion and Prevention (Prevention Act—PrävG)]. http://www.bgbl.de/xaver/bgbl/start.xav?startbk=Bundesanzeiger_BGBl&jumpTo=bgbl115s1368.pdf.

[31] (2018). Institute of Movement Therapy and movement-oriented Prevention and Rehabilitation, German Sport University Cologne. Esports meets Science. https://www.esportwissen.de/en/.

[32] Beckmann K., Glemser A., Heckel C., von der Heyde C., Hoffmeyer-Zlotnik J.H.P., Hanefeld U., Herter-Eschweiler R., Kühnen C. (2016). Demographische Standards. eine gemeinsame Empfehlung des ADM, Arbeitskreis Deutscher Markt- und Sozialforschungsinstitute e.V., der Arbeitsgemeinschaft Sozialwissenschaftlicher Institute e.V. (ASI) und des Statistischen Bundesamtes [Demographical standards—A common recommendation of the ADM working group of German Market. and Social Research Institutes, working group of Social Science Institutes (ASI) and the German Federal Statistical Office].

[33] Chan Y.H. (2003). Biostatistics 104: Correlational analysis. Singap. Med. J..

[34] Game—The German Games Industry Association Nutzer Digitaler Spieler in Deutschland 2018 [Users of Digital Games in Germany 2018]. https://www.game.de/marktdaten/nutzer-digitaler-spiele-in-deutschland-2018/.

[35] Duggan M. Gaming and Gamers. https://www.pewresearch.org/internet/2015/12/15/gaming-and-gamers/.

[36] Wagner M., Hayward I.-C., Wollesen B. (2019). eGaming und psychosoziale Gesundheit [eGaming and psychosocial health]. Interdisziplinäre Forschung und Gesundheitsförderung in Lebenswelten: Bewegung fördern, vernetzen, nachhaltig gestalten [Interdisciplinary Research and Health Promotion in Living Environment: Promoting, Connecting, Designing Sustainable Exercise].

[37] Vermeulen L., van looy J., de Grove F., Courtois C., Authors & Digital Games Research Association DiGRA (2011). You are what you play? A Quantitative Study into game design preferences across gender and their interaction with gaming habits. Proceedings of DiGRA 2011 Conference: Think Design Play.

[38] Lopez-Fernandez O., Williams A.J., Griffiths M.D., Kuss D.J. (2019). Female Gaming, Gaming Addiction, and the Role of Women Within Gaming Culture: A Narrative Literature Review. Front. Psychiatry.

[39] Ruvalcaba O., Shulze J., Kim A., Berzenski S.R., Otten M.P. (2018). Women’s Experiences in eSports: Gendered Differences in Peer and Spectator Feedback During Competitive Video Game Play. J. Sport Soc. Issues.

[40] Lenhart A., Kahne J., Middaugh E., Rankin Macgill A., Evans C., Vitak J. (2008). Teens, Video Games, and Civics. Teens’ Gaming Experiences Are Diverse and Include Significant Social Interaction and Civic Engagement.

[41] Witkowski E. Girl Gamers? Player and Institutional Orientations towards Women’s Participation in and around E-Sports. Presented at Internet Research: The 15th Annual Meeting of the Association of Internet Researchers.

[42] Essential Facts about the Computer and Video Game Industry. https://www.theesa.com/wp-content/uploads/2019/05/2019-Essential-Facts-About-the-Computer-and-Video-Game-Industry.pdf.

[43] Nielsen Sports Esports: Trends & Potenziale [Esports: Trands and potential]. https://nielsensports.com/de/esports-report-deutschland/.

[44] Bonnie R.J., Stroud C., Breiner H. (2015). Investing in the Health and Well-Being of Young Adults.

[45] Ciria L.F., Perakakis P., Luque-Casado A., Morato C., Sanabria D. (2017). The relationship between sustained attention and aerobic fitness in a group of young adults. PeerJ.

[46] Garg M., Lata H., Walia L., Goyal O. (2013). Effect of aerobic exercise on auditory and visual reaction times: A prospective study. Indian J. Physiol. Pharmacol..

[47] Rattray B., Smee D. (2013). Exercise improves reaction time without compromising accuracy in a novel easy-to-administer tablet-based cognitive task. J. Sci. Med. Sport.

[48] (2013). Global Action Plan for the Prevention and Control of Noncommunicable Diseases. 2013–2020.

[49] Warburton D.E.R., Bredin S.S.D. (2017). Health benefits of physical activity: A systematic review of current systematic reviews. Curr. Opin. Cardiol..

[50] Warburton D.E.R., Nicol C.W., Bredin S.S.D. (2006). Health benefits of physical activity: The evidence. CMAJ.

[51] World Health Organization (2010). Global recommendations on physical activity for health.

[52] Finger J.D., Mensink G.B.M., Lange C., Manz K. (2017). Gesundheitsfördernde körperliche Aktivität in der Freizeit bei Erwachsenen in Deutschland [Health-enhancing physical activity during leisure time among adults in Germany]. J. Health Monit..

[53] Dumith S.C., Gigante D.P., Domingues M.R., Kohl H.W. (2011). Physical activity change during adolescence: A systematic review and a pooled analysis. Int. J. Epidemiol..

[54] Sallis J.F. (2000). Age-related decline in physical activity: A synthesis of human and animal studies. Med. Sci. Sports Exerc..

[55] Walch O.J., Cochran A., Forger D.B. (2016). A global quantification of “normal” sleep schedules using smartphone data. Sci. Adv..

[56] Carter B., Rees P., Hale L., Bhattacharjee D., Paradkar M.S. (2016). Association Between Portable Screen-Based Media Device Access or Use and Sleep Outcomes: A Systematic Review and Meta-analysis. JAMA Pediatr..

[57] Cain N., Gradisar M. (2010). Electronic media use and sleep in school-aged children and adolescents: A review. Sleep Med..

[58] Durand D., Landmann N., Piosczyk H., Holz J., Riemann D., Voderholzer U., Nissen C. (2012). Auswirkungen von Medienkonsum auf Schlaf bei Kindern und Jugendlichen [Effects of media use on the sleep of children and adolescents]. Somnologie.

[59] Grønli J., Byrkjedal I.K., Bjorvatn B., Nødtvedt Ø., Hamre B., Pallesen S. (2016). Reading from an iPad or from a book in bed: The impact on human sleep. A randomized controlled crossover trial. Sleep Med..

[60] Chang A.-M., Aeschbach D., Duffy J.F., Czeisler C.A. (2015). Evening use of light-emitting eReaders negatively affects sleep, circadian timing, and next-morning alertness. Proc. Natl. Acad. Sci. USA.

[61] Gradisar M., Wolfson A.R., Harvey A.G., Hale L., Rosenberg R., Czeisler C.A. (2013). The Sleep and Technology Use of Americans: Findings from the National Sleep Foundation’s 2011 Sleep in America Poll. J. Clin. Sleep Med..

[62] Cajochen C., Frey S., Anders D., Späti J., Bues M., Pross A., Mager R., Wirz-Justice A., Stefani O. (2011). Evening exposure to a light-emitting diodes (LED)-backlit computer screen affects circadian physiology and cognitive performance. J. Appl. Physiol..

[63] (2018). DESTATIS—Federal Statistical Office. Mikrozensus—Fragen zur Gesundheit. Körpermaße der Bevölkerung 2017 [Microcensus—questions on health—Body measurements of the population 2017]. https://www.destatis.de/DE/Themen/Gesellschaft-Umwelt/Gesundheit/Gesundheitszustand-Relevantes-Verhalten/Publikationen/Downloads-Gesundheitszustand/koerpermasse-5239003179004.pdf?__blob=publicationFile.

[64] Deutsche Gesellschaft für Ernährung (German Nutrition Society) 10 Regeln für eine vollwertige Ernährung [Ten Rules for a Balanced Nutrition]. https://www.dge.de/fileadmin/public/doc/fm/10-Regeln-der-DGE.pdf.

[65] Mensink G.B.M., Truthmann J., Rabenberg M., Heidemann C., Haftenberger M., Schienkiewitz A., Richter A. (2013). Obst- und Gemüsekonsum in Deutschland: Ergebnisse der Studie zur Gesundheit Erwachsener in Deutschland (DEGS1): [Fruit and vegetable intake in Germany]. Bundesgesundheitsblatt Gesundheitsforschung Gesundheitsschutz.

[66] Shang L., Wang J., O’Loughlin J., Tremblay A., Mathieu M.-È., Henderson M., Gray-Donald K. (2015). Screen time is associated with dietary intake in overweight Canadian children. Prev. Med. Rep..

[67] Newzoo Most Popular Core PC Games | Global. https://newzoo.com/insights/rankings/top-20-core-pc-games/.

[68] Edwards A.L. (1982). The Social Desirability Variable in Personality Assessment and Research, Reprint.

[69] Prince S.A., Adamo K.B., Hamel M.E., Hardt J., Connor Gorber S., Tremblay M. (2008). A comparison of direct versus self-report measures for assessing physical activity in adults: A systematic review. Int. J. Behav. Nutr. Phys. Act..

[70] Dowd K.P., Szeklicki R., Minetto M.A., Murphy M.H., Polito A., Ghigo E., van der Ploeg H., Ekelund U., Maciaszek J., Stemplewski R. (2018). A systematic literature review of reviews on techniques for physical activity measurement in adults: A DEDIPAC study. Int. J. Behav. Nutr. Phys. Act..

[71] Perinelli E., Gremigni P. (2016). Use of Social Desirability Scales in Clinical Psychology: A Systematic Review. J. Clin. Psychol..

