# Supplementary material for: Demographics and Health Behavior of Video Game and eSports Players in Germany: The eSports Study 2019
Source: Int J Environ Res Public Health. 2020 Mar 13;17(6):1870. doi: 10.3390/ijerph17061870 (PMC7142975; doi:10.3390/ijerph17061870)
Supplement: Supplementary file 1 [file ijerph-17-01870-s001.pdf]

Effect sizes:

Kruskal Wallis tests

Age: Statistical significant difference between

- amateurs and occasional players: r=0.15
- amateurs and former professional players: r=0.20
- amateurs and current professional players: r=0.14

Sleep time: Statistical significant difference between

- former and current professional players: r=0.42

ANCOVA

Video game play time: overall partial η^2=0.03

Fisher's exact tests

Gender: Statistical significant difference between

- former professional players and occasional players: φ_c=0.23
- amateurs and regular players: φ_c=0.11
- amateurs and occasional players: φ_c=0.22
- regular players and occasional players: φ_c=0.08

Employment: Statistical significant difference between

- former and current professional players: φ_c=0.42
- current professional players and regular players: φ_c=0.08
- current professional players and occasional players: φ_c=0.25
- former professional players and amateurs: φ_c=0.13
- former professional players and regular players: φ_c=0.09
- amateurs and occasional players: φ_c=0.10

Structured training: Statistical significant difference between

- current professional players and regular players: φ_c=0.21
- former professional players and regular players: φ_c=0.27
- amateurs and regular players: φ_c=0.51
